# Supplementary material for: WearGait-PD: An Open-Access Wearables Dataset for Gait in Parkinson’s Disease and Age-Matched Controls
Source: Sci Data. 2026 Feb 12;13:440. doi: 10.1038/s41597-026-06806-2 (PMC13009270; doi:10.1038/s41597-026-06806-2)
Supplement: Supplementary file 1 — Supplementary Information [file 41597_2026_6806_MOESM1_ESM.docx]

# Supplementary Methods

## Experimental Set-up and Data Collection Approach

The following sensor systems were used during the collection of data for this project. All study sites used the same acquisition software versions to maintain consistency. Some of the supplementary text is duplicated from the main text for completeness.

**Movella/Xsens MTw Awinda Inertial Measurement Units (IMUs) *–*** MTw Awinda is an easy-to-integrate wireless human motion tracker for real-time applications. Thirteen sensors were used in this study, placed at various locations on the body from the head down to the feet. Each sensor is 47 x 30 x 13 mm. All sensors communicate wirelessly with the base station that is connected to a computer running the MT Manager 2019.1.1 acquisition software.

**Moticon OpenGo sensor insoles –** Moticon OpenGo is a wireless in-shoe system with micro-sized, Bluetooth™- and WiFi enabled electronics allowing for quick, natural gait analysis. Six different size inserts were acquired to accommodate different shoe sizes. The sensor insole is intended to be used inside a shoe for measuring the plantar pressure distribution at the sole of foot, and the acceleration and rotation of the foot along three axes. The inserts slip into the shoes of the participant and a mobile app (Version 03.11.00) is used to control data acquisition.

**Protokinetics Pressure walkway *–*** The 16-foot x 2-foot ProtoKinetics Zeno™ Walkway Gait Analysis System detects pressure data during gait, balance, and additional movement protocols. The walkway is equipped with over 18,000 individual sensors that each register 16 different activation levels. Each sensor is 0.5” x 0.5” (1.27 cm x 1.27 cm). The Protokinetics Movement Analysis Software (PKMAS) 6.00c3 was used for data acquisition and raw data export.

**Participant Preparation – IMU** sensors were attached to the participant’s body on the head, xiphoid, the area around the L4/L5 vertebrae, the wrists, thighs, shanks, ankles, and feet. The Moticon OpenGo sensor insoles were placed directly in the shoes. Clinical information was collected and the MDS-UPDRS assessment was performed by a trained specialist on the research team.

**Tasks –** Participants were asked to complete ten tasks in sequence: SelfPace, HurriedPace, SelfPace_mat, HurriedPace_mat, SelfPace_matTURN, TandemGait, Timed Up and Go, Balance, SelfPace_doorpat, and FreeWalk.

## Data Processing

### Data Synchronization and Alignment

After data collection, preprocessing of the walkway data was completed to ensure right and left footfalls were correctly identified. Partial footfalls on the walkway were labeled as ‘Other’. Sensor insole data were also reviewed. Although infrequent, instances of desynchronization can occur during BLE communication between the sensor insoles and the Moticon acquisition App. This may result in asynchronous data capture or prolonged recording from one insole even after the ‘Stop’ command is issued. When such desynchronization is detected, the data from the insoles are adjusted—shifted and/or trimmed—to ensure proper alignment between both insoles and to accurately synchronize the cessation of recording with the conclusion of the data stream. Manual annotation of the participant’s activity using the video data was also completed. Walkway, sensor insole, IMU, and video annotation data were exported and stored in a folder structure with standardized naming conventions.

Due to technical difficulties, some trials required manual alignment between the walkway and IMUs and/or sensor insoles. A list of those participant trials are listed below.
NLS141 – All trials
HC100 – All trials
HC106 – All trials

### Video Annotation

Video recordings—either sagittal view, frontal view, or both—of participants performing walking and balance tasks were collected during all sessions. Upon saving, the videos were deidentified by removing sound and the participant’s face. Frame-by-frame manual annotation of general and clinical events was done by research team members using the MATLAB Video Labeler App (Computer Vision Toolbox). All team members were trained on the definitions prior to performing video annotation. A complete list of general and clinical event definitions is contained in Tables S1 and S2.

Table S1: General Events

| Event |  | Definition |
| --- | --- | --- |
| Sitting | START | The moment when the buttocks comes into contact with the chair. |
|  | STOP | The moment the instructor provides a cue to initiate a task. The end of sitting would correspond to the start of Sit To Stand. |
| SitToStand | START | The moment the “GO” signal is given by the instructor. To ensure this event is consistently annotated, the instructor needs to stand in view of the camera with one of their hands facing the camera in a closed fist. When they say “GO”, they should simultaneously open their fist. |
|  | STOP | The moment of toe off of the first step out from the chair. |
| Standing | START | The moment when both feet are firmly against the floor and no more forward/rotational motion is occurring. |
|  | STOP | The moment of toe-off of the first step OR the moment a ‘Go’ cue is given by the instructor. |
| Walk | START | The moment the “GO” signal is given by the instructor [Gilat 2019]. To ensure this event is consistently annotated, the instructor needs to stand in view of the camera with one of their hands facing the camera in a closed fist. When they say “GO”, they should simultaneously open their fist. |
|  | START for TUG | The moment of toe off of the first step out from the chair. |
|  | END | In most cases, the END of walk will be the start of another event, such as turning or standing. |
| TandemWalk | START | The moment of heel-strike of the first step that starts the tandem gait walk. |
|  | END | The moment of toe off of the first step that intentionally breaks the tandem gait (i.e., when the foot touches down after this toe off, the participant is no longer attempting to walk in a heel-toe pattern). |
|  |  | *Note: turns are not annotated in this task as the tandem walk done prior to the turn is not representative of how a patient would enter a turn |
| Turn | START | The moment of toe-off of the first step that touches down in the area where the turn should be performed with the foot pointing towards the direction of the turn. [Gilat 2019] |
|  | END | Toe off immediately preceding the first large foot swing in the intended direction of travel, where the step length associated with the denoted swing is approaching that seen in the participants full-stride gait. For instances where participants turn to face the direction of travel and briefly pause, the end of walk could be designated as the TO of the first foot swing out of the brief stand if the step length appears reasonable. **For portions of tasks where the subject would not continue moving after a turn (i.e., at the end of a task), the end of a turn could also be designated as the moment that both feet are flat on the ground and the hips and feet are pointing in the same general direction. |
| TurnToSit | START | The moment of toe-off of the first step that touches down in the area where the turn should be performed with the foot pointing towards the direction of the turn. [Gilat 2019] |
|  | END | When the buttocks makes initial contact with the chair. The participant may still be moving after they come in initial contact with the chair. |
| 'EOFeetShoWidth' 'ECFeetShoWidth' 'EOFeetTogether' 'ECFeetTogether' 'EORFootFront' 'EOLFootFront' | START | The moment the participant makes contact with the mat (either foot). (EO = eyes open; EC = eyes closed) |
|  | END | The moment the last foot leaves the mat (either foot). |

Table S2: Clinical Events

| Event |  | Definition |
| --- | --- | --- |
| AttemptsToRise |  | This annotation was included if the subject’s buttocks leaves the chair, but the subject is unable to sustain the rise and falls back into the chair. |
|  | START | The moment the buttocks leaves the chair. |
|  | END | The moment the subject's buttocks comes into contact with the chair after falling back into the chair. |
| UseArmRests |  | Subject requires the use of arm rests to stand from a seated position and/or to sit back down in the chair. |
|  | START | The moment the subject’s places at least one hand/forearm on an arm rest. |
|  | END | The moment the last hand leaves the arm rest. For use of arm rests when sitting back down, the end of UseArmRests should coincide with the start of sitting if the subject has not removed their hand/arm prior to the 'Sitting' start. |
| FoG | START | The moment when the foot of the participant is suddenly no longer producing an effective step forward and is displaying FOG-related features (trembling, shuffling, complete akinesia), despite the participant’s intention to continue walking. [Gilat 2019] |
|  | END | END: The moment of initial toe-off after the FOG when the participant is again able to perform at least two effective alternating steps with both legs showing no FOG-related features. [Gilat 2019] |
| Stagger |  | Subject loses balance thus requiring additional step(s) to the side, front or back to prevent falling. Subject is able to re-capture balance without the assistance of study personnel or objects. |
|  | START | The last contact point of the foot that takes the first unsteady step to the side/front/back. |
|  | END | The first contact point (generally a heel strike, but may be other) of the first footfall after the subject regains control and balance. |

### **FreeWalk Task**

To provide context for the space as participants traverse during the FreeWalk task, an additional ‘Location’ category was added to the annotation. This category includes labels such as Regions of Interest (ROIs), like doors and corners, and Sections that connect these ROIs. Dataset users are encouraged to review the site-specific FreeWalk maps found below to understand the meaning of each ROI and Section label in this task (Figures S1 and S2).

Three events were also added under the ‘General Events’ category. The definitions are below in Table S3.

Table S3: Additional events added to the 'General Event' category during the FreeWalk task

| Stairs | START | Toe off of the foot to make contact with the first step. Continue to label ''Stairs'' until the person completes an ascend and descend, UNLESS the participant takes several steps away from and then back to the stairs. In that case, label a walk-turn-walk sequence that aligns as best as possible with existing definitions. |
| --- | --- | --- |
|  | END | The moment the last foot to leave the step comes in contact with the ground. |
| Chair | START | Loosely defined as the moment the participant begins the process to lower themselves into the chair. |
|  | END | Loosely defined as the moment in which the participant begins advancing forward after rising from the chair. |
| OpenDoor | START | Loosely defined as when the participant starts to move a hand towards the doorknob to open it. |
|  | END | Loosely defined as when the participant lets go of the doorknob. |


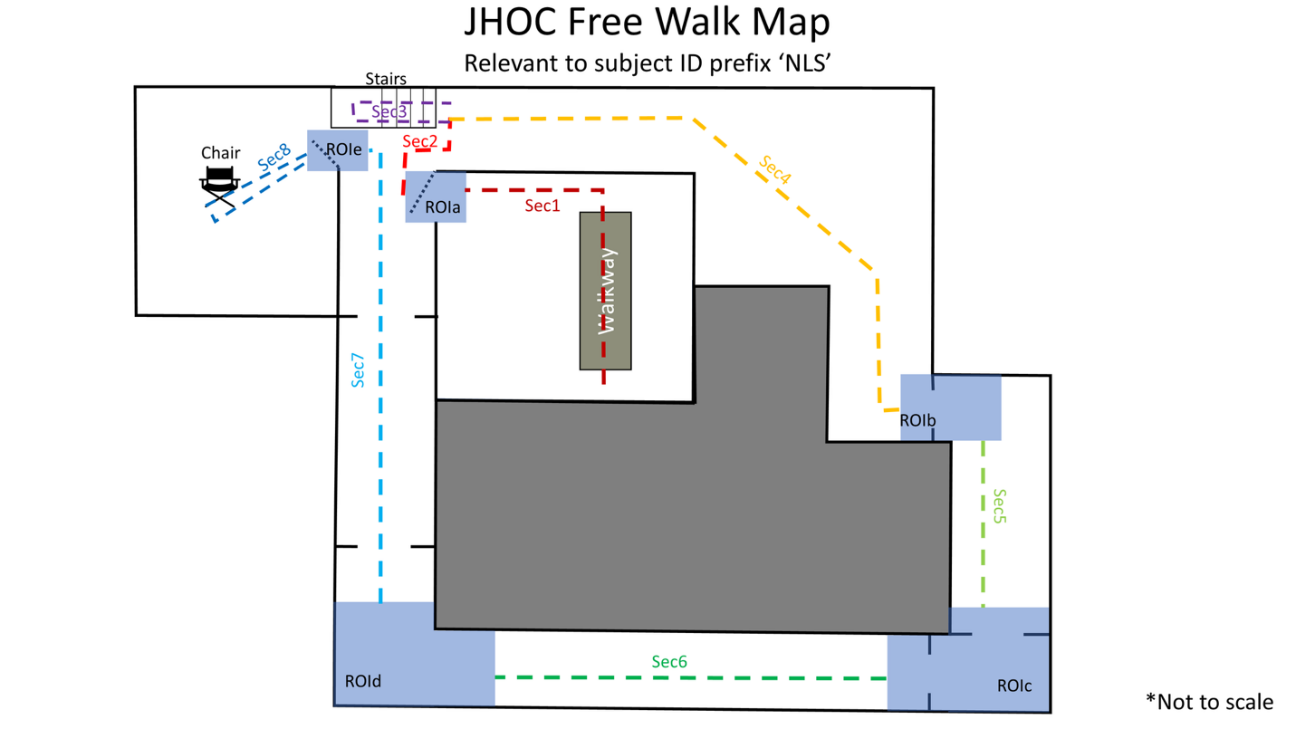


Figure S1: Walking path for the free walk task at the JHOC location.


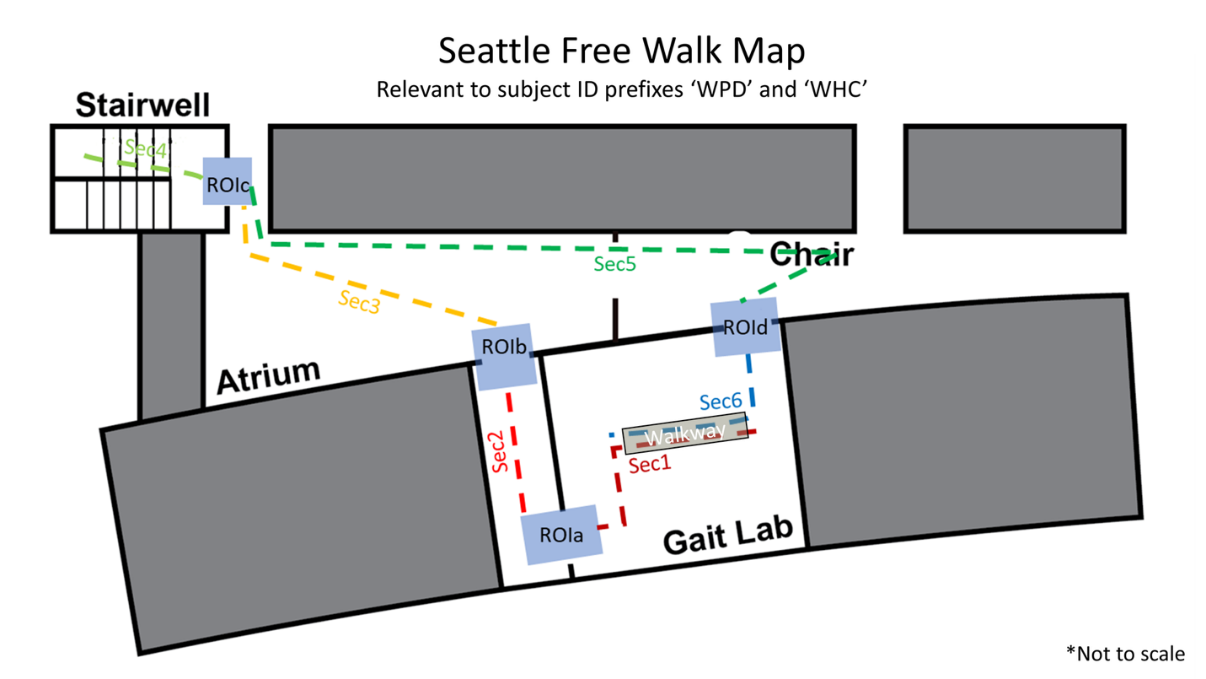


Figure S2: Walking path for the free walk task at the Seattle location.

## Data Quality Assurance and Quality Control

Data quality is of the utmost importance in the curation of an open-access dataset. A quality assurance process was implemented to ensure consistent and accurate data collection. Specifically, a core experimental protocol was developed and reviewed by each researcher. This protocol detailed the steps involved in system-set-up, participant preparation, data collection, and data processing and export. All files were renamed to include the participant ID and task abbreviation immediately after data collection to minimize the risk of file loss due to incorrect folder allocation. For those modalities that required more manual processing (i.e., walkway data and video annotation data), a second researcher reviewed the primary processing completed by the first researcher to ensure proper footfall identification and conformance with the agreed upon annotation event definitions. An Excel spreadsheet accessible to all researchers was also used to track the progression of data processing, from initial collection to MAT and CSV file generation.

After MAT and CSV files were created, a data quality control process on those files was implemented through a series of automatic and manual checks that involved visual inspection of data streams by a researcher to identify data cleanliness and validity issues. Specifically, this process identified issues related to:

- Processing and inclusion of all tasks in the MAT and CSV files
- Integrity of individual IMU sensor data, including the identification of sensor values outside of expected ranges
- Unexpected missing columns of data
- Unexpected large gaps in data
- Expectations around the variable type for a given data variable (e.g., annotations contain no numeric data, IMU data does not contain any errant non-numeric data)
- Video annotation events and expectations surrounding inclusion of specific events for specific tasks
- Alignment of all data, with a particular focus on walkway pressure and sensor insole force data

If an issue was identified, a second researcher was assigned to review and address the issue. Once an issue was addressed, the relevant files were put through the data quality control process again to confirm that all issues were resolved before the final MAT file and final set of CSVs files were generated and included in the dataset.

# Supplementary Notes

## Accessing the data

The WearGait-PD dataset is licensed under the [**Creative Commons Attribution 4.0 International (CC BY 4.0)**](https://creativecommons.org/licenses/by/4.0/legalcode.en) license. Users are free to share and adapt the data, provided you give appropriate credit, provide a link to the license, and indicate if changes were made. To download the data files, users must register for a Synapse account. When registering, users must review and agree to the Synapse governance policies and procedures (Terms and Conditions of Use, Privacy Policy, Awareness and Ethics Pledge). You can review the Synapse Governance documents [**here**](https://help.synapse.org/docs/Synapse-Governance.2004255211.html).

## Data description

Version 1 of the dataset can be accessed at: https://www.synapse.org/Synapse:syn55052683

All clinical and demographic information available for all [PD patients](https://www.synapse.org/Synapse:syn55105530) and [control participants](https://www.synapse.org/Synapse:syn55105521) can be found in CSV spreadsheets within the Version1 data folder at the above link. In these demographics + clinical spreadsheets, each row constitutes one participant, with the first column listing the participant ID. All participant IDs are alphanumeric, with the letters denoting the study site at which the participant was recorded. The prefix NLS is associated with the JHOC site, prefix HC is associated with the JHM Bayview site, and prefixes WPD and WHC are associated with the VA-Seattle site. All columns are labeled with the variable name.

Formatting scripts were written to align all wearables and reference data and save data as MAT files and comma-separated value (CSV) files. For a given participant, there are 8 CSV files (1 file/task) that represent the annotation events, walkway data, insole data, and IMU data. These data can be found in a folder labeled with the participant ID. The same data for each participant is also represented as one MAT file that contains 8 structure variables for each task, with each task containing 8 fields (Table S4).

Table S4: Field names and descriptions contained with each MAT file

| Annotation: | tx2 timetable containing video annotation information (tx3 for FreeWalk task) |
| --- | --- |
| Walkway: | tx8 timetable containing sensor and contact walkway data |
| Insole: | tx38 timetable containing insole data |
| IMU_acc: | tx84 timetable containing Acc and FreeAcc variables for each IMU and insole |
| IMU_gyr: | tx45 timetable containing gyroscope data for each IMU and insole |
| IMU_mag: | tx39 timetable containing magnetometer data for each IMU |
| IMU_velInc: | tx39 timetable containing Δv data for each IMU |
| IMU_orient: | tx91 timetable containing Δq and roll, pitch, yaw data for each IMU |

All data were collected using a sampling frequency of 100 Hz. For missing numeric data due to connectivity issues, sensor malfunctions, or lack of instrumentation for a given participant/task, NaN values are used. [Note: the data alignment process also used NaN values at the beginning and/or end to pad the time series, so NaN values in these locations are expected. Sensors not available during a given data collection are excluded from CSV output files but represented as NaN columns in the MAT files.] For the GeneralEvent and ClinicalEvent variables containing frame-by-frame annotations, the string “unlabeled” was used to represent portions of a trial that did not match any annotation category (e.g., shuffling at the end of a trial instead of standing). A description of all the variables within each field in the MAT file is provided below (Table S5). These variable names are the same within the CSV files.

Table S5: A description of all the variables within the CSV file and each field in the MAT file.

| **MAT Field name** | **Variable name** | **Description** | **Unit** |
| --- | --- | --- | --- |
| Annotation | GeneralEvent | Annotation of a general event | N/A |
| Annotation | ClinicalEvent | Annotation of a clinical event | N/A |
| Walkway | LFootContact | Binary marker of left foot contact (partial footfalls excluded*) | 0 means no contact; 1 means contact |
| Walkway | RFootContact | Binary marker of right foot contact (partial footfalls excluded*) | 0 means no contact; 1 means contact |
| Walkway | LFootPressure | Relative pressure of left foot | arbitrary units (a.u.) |
| Walkway | RFootPressure | Relative pressure of right foot | a.u. |
| Walkway | Walkway_X | Vector of x-coordinates for active walkway sensors at time point t, where x_i_ corresponds with y_i_. [sensor dimensions are 0.5” x 0.5” or 1.27 cm x 1.27 cm] | x-coordinate |
| Walkway | Walkway_Y | Vector of y-coordinates for active walkway sensors at time point t, where x_i_ corresponds with y_i_. [sensor dimensions are 0.5” x 0.5” or 1.27 cm x 1.27 cm] | y-coordinate |
| Walkway | WalkwayPressure | Walkway pressures for each sensor | Activation level (0-15 binned levels) |
| Walkway | WalkwayFoot | Detection of foot at each sensor | L/R/other |
| IMU_acc | [XX]*Acc*X | Acceleration of the IMU [XX] in the X dimension | m/s^2^ |
| IMU_acc | [XX]*Acc*Y | Acceleration of the IMU [XX] in the Y dimension | m/s^2^ |
| IMU_acc | [XX]*Acc*Z | Acceleration of the IMU [XX] in the Z dimension | m/s^2^ |
| IMU_acc | [XX]*FreeAcc*E | Acceleration in the [XX] local earth-fixed reference frame with local gravity deducted, east | m/s^2^ |
| IMU_acc | [XX]*FreeAcc*N | Acceleration in the [XX] local earth-fixed reference frame with local gravity deducted, north | m/s^2^ |
| IMU_acc | [XX]*FreeAcc*U | Acceleration in the [XX] local earth-fixed reference frame with local gravity deducted, up | m/s^2^ |
| IMU_gyr | [XX]*Gyr*X | 3D rate of turn in X dimension in IMU [XX] | rad/s |
| IMU_gyr | [XX]*Gyr*Y | 3D rate of turn in Y dimension in IMU [XX] | rad/s |
| IMU_gyr | [XX]*Gyr*Z | 3D rate of turn in Z dimension in IMU [XX] | rad/s |
| IMU_mag | [XX]*Mag*X | Magnetic field in X dimension in IMU [XX] | a.u. |
| IMU_mag | [XX]*Mag*Y | Magnetic field in Y dimension in IMU [XX] | a.u. |
| IMU_mag | [XX]*Mag*Z | Magnetic field in Z dimension in IMU [XX] | a.u. |
| IMU_velInc | [XX]*VelInc*X | Velocity increment from strap-down integration (SDI) algorithm in X dimension in IMU [XX] | m/s |
| IMU_velInc | [XX]*VelInc*Y | Velocity increment from SDI algorithm in Y dimension in IMU [XX] | m/s |
| IMU_velInc | [XX]*VelInc*Z | Velocity increment from SDI algorithm in Z dimension in IMU [XX] | m/s |
| IMU_orient | [XX]*OriInc*q0 | Orientation increment quaternion from SDI in IMU [XX], q0, where delta_q is the incremental quaternion that represents the change in orientation during a specific time interval | a.u. |
| IMU_orient | [XX]*OriInc*q1 | Orientation increment quaternion from SDI in IMU [XX], q1, where delta_q is the incremental quaternion that represents the change in orientation during a specific time interval | a.u. |
| IMU_orient | [XX]*OriInc*q2 | Orientation increment quaternion from SDI in IMU [XX], q2, where delta_q is the incremental quaternion that represents the change in orientation during a specific time interval | a.u. |
| IMU_orient | [XX]*OriInc*q3 | Orientation increment quaternion from SDI in IMU [XX], q3, where delta_q is the incremental quaternion that represents the change in orientation during a specific time interval | a.u. |
| IMU_orient | [XX]_Roll | Roll in IMU [XX] using Euler angle convention XYZ with Earth fixed type | degree |
| IMU_orient | [XX]_Pitch | Pitch in IMU [XX] using Euler angle convention XYZ with Earth fixed type | degree |
| IMU_orient | [XX]_Yaw | Yaw in IMU [XX] using Euler angle convention XYZ with Earth fixed type | degree |
| Insole | LPressure[YY] | Pressure in sensor [YY] of the left insole | N/cm^2^ |
| IMU_acc | LinsoleAcc_X | Acceleration in X direction of the left insole | m/s^2^ |
| IMU_acc | LinsoleAcc_Y | Acceleration in Y direction of the left insole | m/s^2^ |
| IMU_acc | LinsoleAcc_Z | Acceleration in Z direction of the left insole | m/s^2^ |
| IMU_gyr | LinsoleGyr_X | Angular Velocity in X direction of the left insole | rad/s |
| IMU_gyr | LinsoleGyr_Y | Angular Velocity in Y direction of the left insole | rad/s |
| IMU_gyr | LinsoleGyr_Z | Angular Velocity in Z direction of the left insole | rad/s |
| Insole | LTotalForce | Total Force on the left insole | N |
| Insole | LCoP_X | Center of pressure in X direction of the left insole, normalized | [-0.5,0.5] |
| Insole | LCoP_Y | Center of pressure in Y direction of the left insole, normalized | [-0.5,0.5] |
| Insole | RPressure[YY] | Pressure in sensor [YY] of the right insole | N/cm^2^ |
| IMU_acc | RinsoleAcc_X | Acceleration in X direction of the right insole | m/s^2^ |
| IMU_acc | RinsoleAcc_Y | Acceleration in Y direction of the right insole | m/s^2^ |
| IMU_acc | RinsoleAcc_Z | Acceleration in Z direction of the right insole | m/s^2^ |
| IMU_gyr | RinsoleGyr_X | Angular Velocity in X direction of the right insole | rad/s |
| IMU_gyr | RinsoleGyr_Y | Angular Velocity in Y direction of the right insole | rad/s |
| IMU_gyr | RinsoleGyr_Z | Angular Velocity in Z direction of the right insole | rad/s |
| Insole | RTotalForce | Total Force on the right insole | N |
| Insole | RCoP_X | Center of pressure in X direction of the right insole, normalized | [-0.5,0.5] |
| Insole | RCoP_Y | Center of pressure in Y direction of the right insole, normalized | [-0.5,0.5] |

* Partial footfalls, defined as footfalls that occurred with a portion of the foot off the walkway such that heel strike and toe off could not be identified, were labeled as ‘Other’ during pre-processing.

In the table above, [XX] denotes IMU location and can be one of the following:

- LowerBack
- R_DorsalFoot
- L_DorsalFoot
- R_Wrist
- L_Wrist
- R_Ankle
- L_Ankle
- R_MidLatThigh
- L_MidLatThigh
- R_LatShank
- L_LatShank
- Xiphoid
- Forehead

## Sensor Coordinate Systems

The figure below shows the sensor-fixed coordinate system for the IMU sensors (Figure S3).


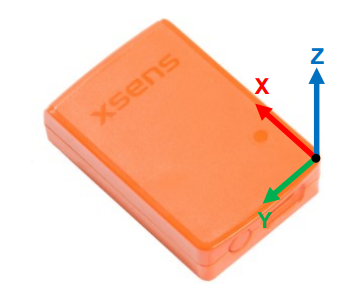


Figure S3: Sensor-fixed coordinate system for the IMU sensors

Free acceleration (FreeAcc) is the acceleration in the local earth-fixed reference coordinate system from which the local gravity is deducted. By default, the local earth-fixed reference coordinate system used is defined as a right-handed Cartesian coordinate system with X positive to the East (E), Y positive to the North (N), and Z positive when pointing up (U).

[YY] denotes pressure sensor 1-16 as identified in Figure S4 below (the right insole sensor layout mirrors that of the left). Location/orientation of the accelerometer and gyroscope is also depicted.


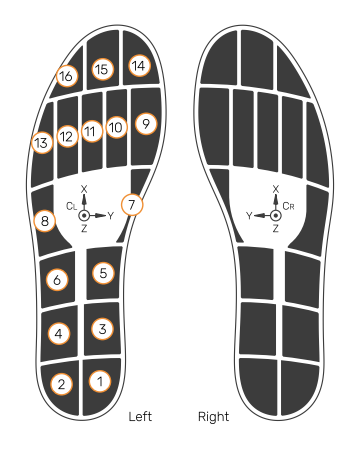


Figure S4: Location of individual pressure sensors,

accelerometer, and gyroscope in the sensor insoles.

Figure S5 shows the coordinate system for the sensor activation data from the walkway.


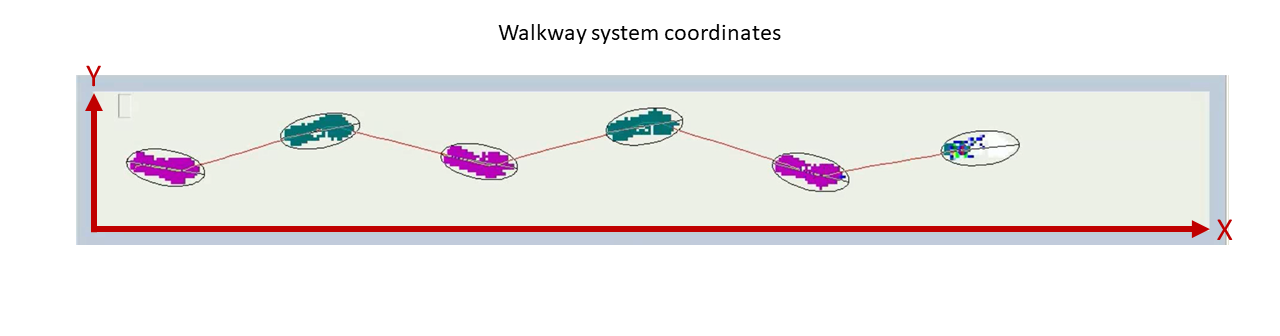


Figure S5: Coordinate system for the sensor activation data from the walkway.

## Sensor Insole Synchronization Characterization

The pressure sensing walkway provides the primary synchronization clock for this dataset. For the sensor insoles, data recording is initiated manually with the Moticon OpenGo App on either a tablet or phone with an Android operating system. The Android device communicates with a custom time synchronization box capable of sending a TTL pulse that can be measured and logged by the walkway acquisition software. During a trial, data recording was manually initiated on the sensor insole app after recording started on the walkway so that the walkway could log the TTL pulse, and this signal was used to align the walkway and sensor insole data during post-processing.

During pilot work, visual inspection showed that the method of synchronizing the sensor insoles results in a time delay in the sensor insole signals relative to the walkway signal. Figure S6 shows normalized walkway pressure activation and insole forces for the right foot during a task as an example of the observed delay.


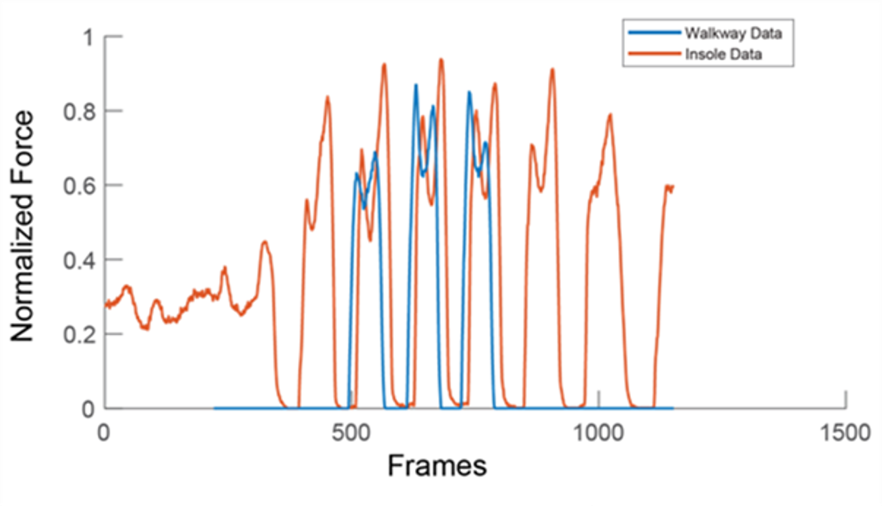


Figure S6: Normalized pressure walkway activations and total insole force for the

right foot during ambulation across the walkway, showing a consistent time delay.

Troubleshooting indicated this delay was likely due to internal delays between when data recording was initiated in the Android app and when a TTL pulse was sent to the walkway.

Without the ability to interrogate the Moticon OpenGo App, it was determined that this delay would be corrected most efficiently in post-processing, when data from all the systems are imported and aligned. Benchtop experiments and analyses were conducted to assess consistency of the delay across data collection sites, sensor insole sizes, and data collection sessions. The purpose of this page is to describe the benchtop test methods and analyses used to characterize the time delay in the sensor insole system at each site.

***Benchtop Test Methods***

Initially, walking data were used to characterize the delay between the walkway and insole waveforms. However, after pilot analyses, it was determined these data were not adequate for characterization of this delay. Both sensing modalities employ one-dimensional pressure sensors, but the foot and insole change orientation across the gait cycle, with the result that vertical and shear loads in the lab coordinate system are measured differently between the two systems. This leads to true differences in the measured waveform shapes between the insoles and walkway, which complicates any estimate of delay between the two.

Therefore, a controlled method of applying consistent, repeatable loading waveforms that were distinctly present in both data streams was developed, facilitating their temporal alignment and characterization of potential variances. During benchtop tests, sensor insoles were placed inside of a shoe on a prosthetic foot. The prosthetic foot was mounted to the end of a crutch and was vertically pressed into the walkway during tests. During a test, the experimenters initialized data collection on both systems using the typical data collection protocol, and one of the experimenters loaded the prosthesis and crutch with 25-50% of their bodyweight. A single trial consisted of ten independent loading cycles, where the prosthesis was lifted completely off the walkway between presses. The experimenter loaded the prosthesis as vertically as possible to avoid shear loading.

At each data collection site, thirty unique trials were recorded over two data collection sessions. At the VA - Seattle site, the data collection sessions were roughly two weeks apart. At the Johns Hopkins Outpatient Center and Bayview campus data collection sites, the two data collection sessions were on the same day, but the hardware was power-cycled between sessions. During the first session, five trials of ten loading waveforms for both the size five and size six insoles were collected. During the second session, five trials of ten loading waveforms for sizes four, five, six, and seven were collected. Overall, thirty trials with 300 unique loading events were collected at each site.

***Delay Characterization and Analysis***

An algorithm was employed to compute the sensor insole delay relative to the walkway for each unique trial. The high-level steps of the delay characterization analysis were as follows:

1. All insole data were preprocessed by filling short sections of missing data with a spline filter.
2. The delay for a given trial was estimated by determining what amount of delay correction (i.e., shift of the insole signal backwards in time) maximized [**cross-correlation**](https://www.mathworks.com/help/signal/ref/alignsignals.html) between the insole ‘Total Force’ and walkway ‘Total Pressure’ waveforms.

After trial-by-trial delay estimates were computed for all trials, summary statistics were computed to describe the mean, standard deviation, and maximum/minimum values of the delay. Data from each site were analyzed independently, as there are minor variations in the insole hardware at each site. To determine if insole size and data collection session affected delay estimates, a two-way analysis of variance (ANOVA) was conducted with size and session as independent factors.

***Results***

VA-Seattle Site
In Seattle, the mean delay across all 30 trials was 11.1 ± 0.84 samples. The trial with the maximum delay required a 13-sample shift, and the trial with the minimum delay required a 9-sample shift. Rounding to the nearest integer, the Seattle average delay in the insole data is 11 samples. Simple main effects analysis from the two-way ANOVA revealed that insole size (F = 0.73, df = 3, p=0.55) and data collection session (F = 0.28, df = 1, p=0.60) did not have a statistically significant effect on delay estimation.

Johns Hopkins Outpatient Center (JHOC) Site
At JHOC, the mean delay across all 30 trials was 13.2 ± 0.92 samples. The trial with the maximum delay required a 15-sample shift, and the trial with the minimum delay required an 11-sample shift. Rounding to the nearest integer, the JHOC delay in the insole data is 13 samples. Simple main effects analysis from the two-way ANOVA revealed that insole size (F = 0.92, df = 3, p=0.45) and data collection session (F =0.92, df = 1, p=0.35) did not have a statistically significant effect on delay estimation.

Johns Hopkins Bayview Site
At Bayview, the mean delay across all 30 trials was 9.97 ± 0.93 samples. The trial with the maximum delay required an 11-sample shift, and the trial with the minimum delay required an 8-sample shift. Rounding to the nearest integer, the JHOC delay in the insole data is 10 samples Simple main effects analysis from the two-way ANOVA revealed that insole size (F = 2.49, df = 3, p=0.08) and data collection session (F = 0.27, df = 1, p=0.61) did not have a statistically significant effect on delay estimation.

Figure S7 below shows an example section of one of the testing waveforms from the VA-Seattle site before (top plot) and after (bottom plot) a 10-sample shift was applied to the insole data. These results are representative of the alignment observed across all study sites.


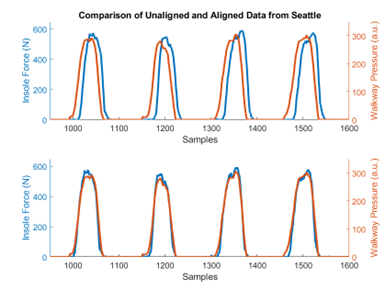


Figure S7: Example trajectories from the VA-Seattle site of the walkway pressure and sensor

insole force before (top) and after (bottom) a 10-sample shift was applied to the insole data.

***Summary***

The site-specific delay corrections for Seattle, JHOC, and Bayview were 11 samples, 13 samples, and 10 samples, respectively. Estimates from each site had sub-sample standard deviations, and neither insole size nor data collection session had statistically significant effects on delay. Overall, these findings support applying the consistent, site-specific delays to the insole data during the initial import and alignment processing scripts.

It is likely the delay is caused by factors within the Moticon OpenGo App, the Android devices that run the app, the custom synchronization hardware, or a combination of the three. Thus, it is difficult to guarantee these delays will not change over time with, e.g., a required software update. Due to this uncertainty, additional quality checks were incorporated within the data import and alignment scripts. These quality checks monitor and log the cross-correlation of the walking data waveforms over time and raise warnings if the cross-correlation values decrease beyond a preset threshold. In that event, new benchtop data will be collected to determine adjusted delay corrections for each site.
